# Concurrent Bilateral Anterior Tibial Stress Fractures and Vitamin D Deficiency in an Adolescent Female Athlete: Treatment With Early Surgical Intervention

**DOI:** 10.3389/fped.2019.00397

**Published:** 2019-10-04

**Authors:** Jane S. Chung, Meagan J. Sabatino, Amanda L. Fletcher, Henry Bone Ellis

**Affiliations:** ^1^Texas Scottish Rite Hospital for Children, Dallas, TX, United States; ^2^University of Texas Southwestern Medical Center, Dallas, TX, United States

**Keywords:** anterior tibial diaphyseal stress fractures, intramedullary nailing, vitamin D deficiency, high risk stress fractures, adolescent athlete, bone health

## Abstract

**Case:** A 16-year-old African American multi-sport female athlete presents with bilateral worsening activity-related leg pain for 5 months. Multiple bilateral anterior tibial diaphyseal stress fractures and significant vitamin D deficiency were identified. She was treated with a combination of vitamin D supplements and static intramedullary nailing of the bilateral tibias resulting in clinical and radiographic healing and return to sports.

**Discussion:** Vitamin D deficiency and high level of activity in a young athlete may be the etiology to atypical multiple stress fractures. In athletes who may want to return to sport rapidly, early operative intervention and correction of vitamin D deficiency may be treatment options.

**Level of Evidence:** Level V- case report.

## Background

Anterior tibial diaphyseal stress fractures are commonly seen in runners and dancers and are considered “high risk” stress fractures due to prolonged healing (1). These injuries can appear as the “dreaded black line” over the anterior tibial cortex; however, plain radiographs have been shown to have a low sensitivity (10–50%) for detecting stress fractures, particularly if presented early on in the clinical course (2). Although a trial of conservative management is initially recommended, early surgical intervention may be considered in patients with risk factors associated with delayed healing (i.e., metabolic or nutritional concerns). Consideration for investigation into the etiology of stress fracture beyond activity is needed in atypical presentations.

The physician must take into consideration the characteristics of the stress fracture as well as the athlete's level of sport and athletic timeline. One benefit of early surgical intervention is a faster return to sport, with a mean timeline of 4 months (3) compared to 6–12 months with conservative management. Current surgical options include IM nailing, tension band plating, and techniques, such as drilling and debridement of the fracture site with bone grafting (1, 4–6). Currently, no specific guidelines for managing anterior tibial diaphyseal stress fractures exist, and these must be treated on a case-by-case basis.

## Informed Consent

Written informed consent for the publication of this case report was waived by the University of Texas Southwestern Medical Center Institutional Review Board. In place, verbal consent from the patient and guardian were obtained for the publication of the case report.

## Case Report

A 16 year and 7-month-old African American female, who participates in multiple sports including cheer, softball, and competitive volleyball, presents with 5 months of worsening atraumatic bilateral anterior leg pain. She normally trains 6 h per day and 4 days per week. Initially, her pain only occurred with sports-related activities; however, after a recent 3-day volleyball tournament, her pain acutely worsened, yet improved with rest.

She denied prior history of stress fractures, multiple previous fractures, and a family history of bone diseases, such as osteogenesis imperfecta. She is otherwise healthy with menarche at age 11 and reported normal cycles. The mother reported the patient had no dietary concerns but could be eating healthier. The patient had a normal BMI, with no concerning signs of metabolic or hormonal abnormalities.

**Figure 1:**
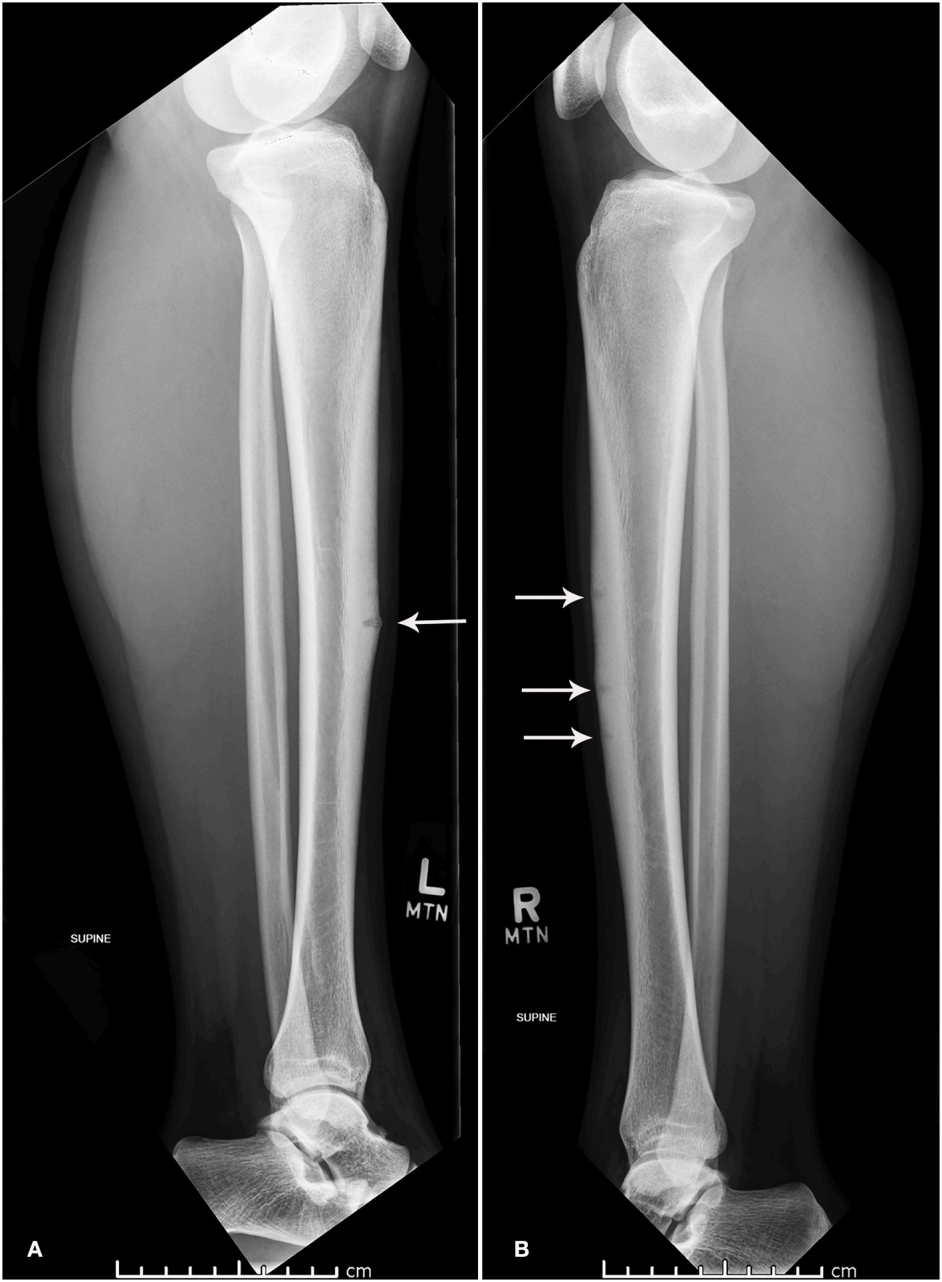
Lateral x-ray of left **(A)** and right **(B)** tibia and fibula.

After obtaining radiographs and a physical exam, the patient was found to have bilateral multiple anterior cortex mid-tibial diaphyseal stress fractures. Three focal lucencies were noted over the anterior cortex of the right mid-tibial diaphysis and one over the left with bilateral cortical thickening and periosteal reaction (Figure 1).

Bone metabolic labs and bilateral lower extremity CT scans were obtained. The patient's serum calcium was normal at 9.4 mg/dL, but her 25-hydroxy vitamin D resulted low at 17 ng/mL, and she was diagnosed with vitamin D deficiency. All other labs were noted to be within normal limits. She was started on high-dose vitamin D at 50,000 IU weekly for 8 weeks, and was referred to a registered dietician for consultation. CT scan of the bilateral lower extremities demonstrated additional smaller lucent defect in the left anterior cortex proximal to the stress fracture noted on x-ray.

**Figure 2:**
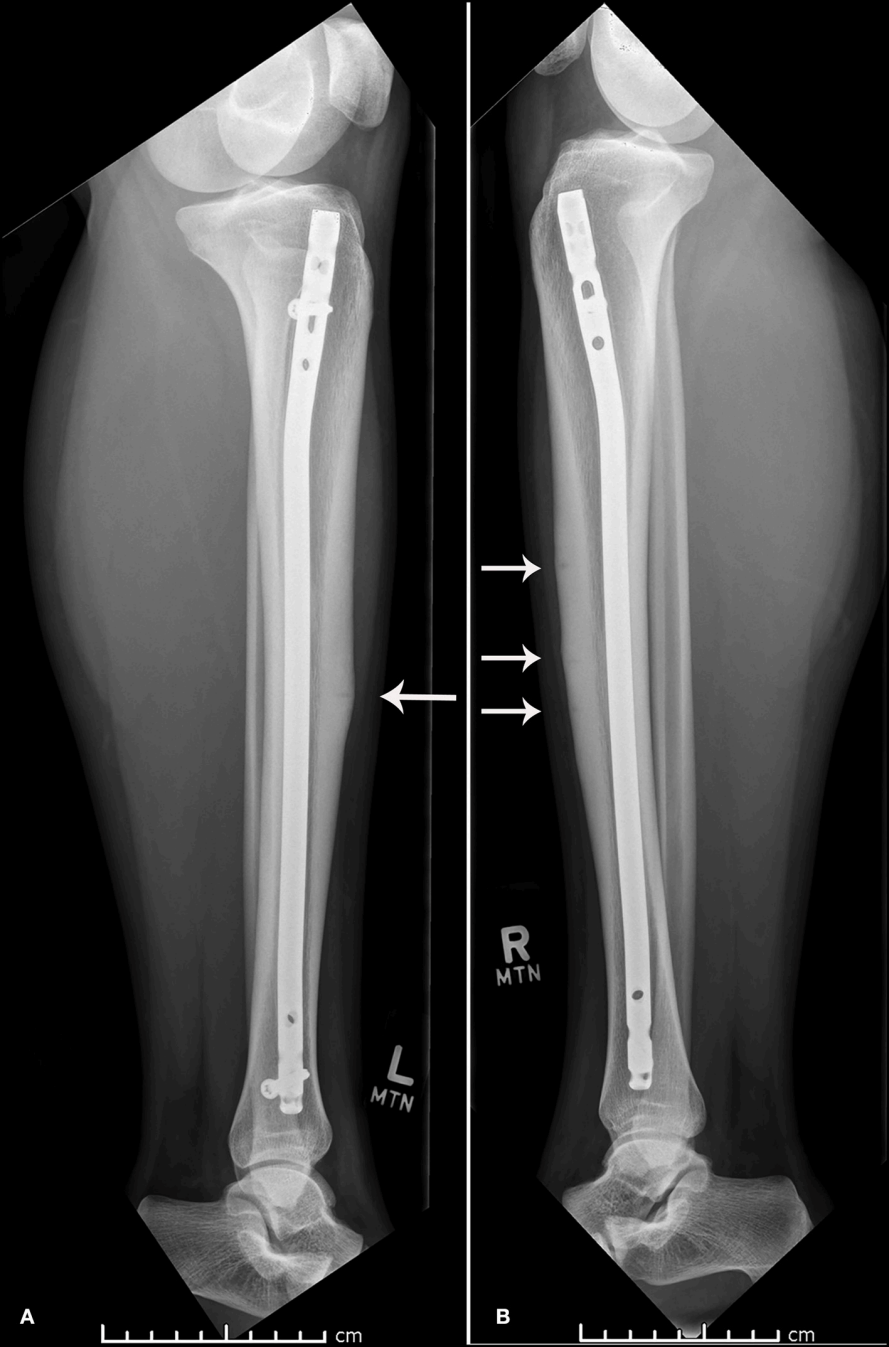
One year post-operative lateral x-ray of the left **(A)** and right **(B)** tibia and fibula.

The atypical nature of multiple stress fractures and a low vitamin D level were concerning for possible prolonged healing. After extensive discussion with the patient and parents regarding activity level and risks of operative management, they wished to proceed with surgery for a potentially faster return to competitive volleyball in hopes of obtaining collegiate scholarships. She first underwent a transpatellar tendon reamed intramedullary nailing of the more symptomatic left tibia with proximal and distal locking screws (7). She underwent IM nailing of the contralateral tibia 6 weeks later. She attended physical therapy shortly after the right tibial procedure, focusing on a sport-specific return to play. Three and a half months after surgery, she reported the pain had dramatically improved and was cleared to gradually return to sports. Improvement in radiographic appearance of the linear lucencies was appreciated. At the 1-year post-operative follow-up, the patient had returned to full sports (HSS Pedi-FABS = 23) and reported minimal anterior knee pain with impact-related activities. She transitioned her vitamin D supplements to 1,000 IU QD, and her most recent 25-hydroxy vitamin D level was noted to be normal at 41 ng/mL (Figure 2).

## Discussion

Multiple stress fractures of the anterior tibial cortex involving bilateral lower extremities are not common in pediatric athletes. There has been a case report of a 21-year-old football athlete with multiple single extremity anterior tibial cortex stress fractures, who later developed a tibial stress fracture on the contralateral side (8). The patient failed conservative management and was treated operatively with IM nailing.

Tibial stress fractures account for 26–40% of all stress fractures, most commonly in distance runners (5). The three most common sites for tibial stress fractures are posteromedial cortex (most common), tibial plateau, and anterior cortex. The anterior cortex (tension side of tibia) is affected in about 5% of these (9). Stress fractures involving the anterior cortex were first mentioned by Burrows in 1956, describing the lesion in five ballet dancers (10). While most tibial stress fractures tend to heal with conservative management, those that involve the anterior cortex are at increased risk for prolonged recovery, delayed healing, non-union, or even complete fracture.

There are multiple factors that can predispose an athlete to developing stress fractures. Examples of intrinsic factors include metabolic bone diseases and nutritional or hormonal deficiencies. In particular, young female athletes with a low BMI and history of amenorrhea are at increased risk for stress fractures and should be screened for possible female athlete triad (11). Extrinsic factors include type of sport, training surface, shoe wear, and most commonly a sudden increase in training regimen (12). This patient participated in moderate intensity sports without a significant change in activity and was found to have a significant underlying vitamin D deficiency. Vitamin D deficiency has been reported in the literature in patients with lower extremity stress fractures and should be evaluated if clinical concern warrants (13, 14). Diet, genetics, and participation in weight-bearing activities influence bone mass accrual in the pediatric population, with about 90% of adult bone mass being acquired during adolescence (15, 16) Diet and nutrition are important considerations for bone health and fracture prevention. Increased consumption of calcium, vitamin D, and protein may play a preventative role against stress fracture development. (5, 6, 14, 17–20) Prospective studies including only females showed that increased intake of calcium and vitamin D supplements, dietary calcium, or dairy products (calcium, vitamin D, and protein) were associated with decreased incidence of stress fractures (13, 21, 22).

Currently there are no specific guidelines for managing anterior tibial diaphyseal stress fractures (1). The role of initial surgery is unclear. A trial of conservative management is typically advocated for the first 3–6 months, including rest, modified weight-bearing, activity modification, LIPUS (low-intensity pulsed ultrasound), and ECST (extracorpeal shockwave therapy) (3, 23, 24). Surgical intervention is recommended for persistent symptoms, delayed healing or non-union; however, in the high-level athlete, the benefits of early return to sports with surgical intervention can be considered. Tibial stress fractures, when treated conservatively, may take more than 12 months for healing, as compared to an 11 weeks to 4 month return to sport with tension band plating or IM nailing of the tibia (4, 10, 25). One study looking at 50 patients with anterior tibial stress fractures treated conservatively revealed only 40% successfully returning to full activity (26).

Described surgical treatment options for anterior mid-shaft tibial stress fractures include drilling (9), excision/grafting (9), tension plating (25), or IM fixation (3, 27). Intramedullary fixation has been advocated with good to excellent results; however, between 47.4 and 73.2% of patients may have some form of residual knee pain (28, 29). Tension band plating has been an option to avoid anterior knee pain in high-level athletes; however, it is not indicated when multiple lucencies are identified. In this case, the anterior tibia pain resolved with some residual anterior knee pain complaints, relieved with patella mobilization and stretching.

There is lack of literature supporting initial surgical treatment of anterior tibial cortex stress fractures, hence, comparing surgical and conservative treatment is difficult. For this patient, 1 year post-operative x-rays revealed some remaining signs of anterior tibial cortex lucency, showing us that these would likely still not have healed with conservative management. Although she has reported full return to activity, she does continue to report intermittent activity- related pain believed to be patellofemoral in nature, which is relieved by topical NSAIDs.

When treating young pediatric athletes with multiple stress fractures of the anterior tibia, it is important that the clinician considers the goals of the athlete to best recommend conservative vs. early operative management. Suspicions for other intrinsic factors that contribute to multiple stress fractures, including poor metabolic bone health, should be investigated. Calcium and vitamin D may play an important role in the prevention of stress fractures; however, more prospective studies are needed to evaluate this in pediatric athletes.

## Data Availability Statement

All datasets generated for this study are included in the manuscript/supplementary files.

## Ethics Statement

The need for ethical approval for this study was waived by UT Southwestern Human Research Protection Program.

## Author Contributions

All authors have provided substantial contributions to the conception or design of the work. All authors have also drafted the work or revised it critically for important intellectual content, and have given final approval of the version to be published, and are in agreement to be accountable for all aspects of the work in ensuring that questions related to the accuracy or integrity of any part of the work are appropriately investigated and resolved.

### Conflict of Interest

The authors declare that the research was conducted in the absence of any commercial or financial relationships that could be construed as a potential conflict of interest.
